# Predicting mortality and hospitalization in heart failure using machine learning: A systematic literature review

**DOI:** 10.1016/j.ijcha.2021.100773

**Published:** 2021-04-12

**Authors:** Dineo Mpanya, Turgay Celik, Eric Klug, Hopewell Ntsinjana

**Affiliations:** a Division of Cardiology, Department of Internal Medicine, School of Clinical Medicine, Faculty of Health Sciences, University of the Witwatersrand, Johannesburg, South Africa; b School of Electrical and Information Engineering, Faculty of Engineering and Built Environment, University of the Witwatersrand, Johannesburg, South Africa; c Netcare Sunninghill, Sunward Park Hospitals and Division of Cardiology, Department of Internal Medicine, School of Clinical Medicine, Faculty of Health Sciences, University of the Witwatersrand and the Charlotte Maxeke Johannesburg Academic Hospital, Johannesburg, South Africa; d Department of Paediatrics and Child Health, School of Clinical Medicine, Faculty of Health Sciences, University of the Witwatersrand, Johannesburg, South Africa; e Wits Institute of Data Science, University of the Witwatersrand, Johannesburg, South Africa

**Keywords:** Heart failure, Risk score, Predictive modelling, Machine learning, Sub-Saharan Africa, Mortality, Hospitalization

## Abstract

**Objective:**

The partnership between humans and machines can enhance clinical decisions accuracy, leading to improved patient outcomes. Despite this, the application of machine learning techniques in the healthcare sector, particularly in guiding heart failure patient management, remains unpopular. This systematic review aims to identify factors restricting the integration of machine learning derived risk scores into clinical practice when treating adults with acute and chronic heart failure.

**Methods:**

Four academic research databases and Google Scholar were searched to identify original research studies where heart failure patient data was used to build models predicting all-cause mortality, cardiac death, all-cause and heart failure-related hospitalization.

**Results:**

Thirty studies met the inclusion criteria. The selected studies' sample size ranged between 71 and 716 790 patients, and the median age was 72.1 (interquartile range: 61.1–76.8) years. The minimum and maximum area under the receiver operating characteristic curve (AUC) for models predicting mortality were 0.48 and 0.92, respectively. Models predicting hospitalization had an AUC of 0.47 to 0.84. Nineteen studies (63%) used logistic regression, 53% random forests, and 37% of studies used decision trees to build predictive models. None of the models were built or externally validated using data originating from Africa or the Middle-East.

**Conclusions:**

The variation in the aetiologies of heart failure, limited access to structured health data, distrust in machine learning techniques among clinicians and the modest accuracy of existing predictive models are some of the factors precluding the widespread use of machine learning derived risk calculators.

## Graphical abstract

**Figure:**
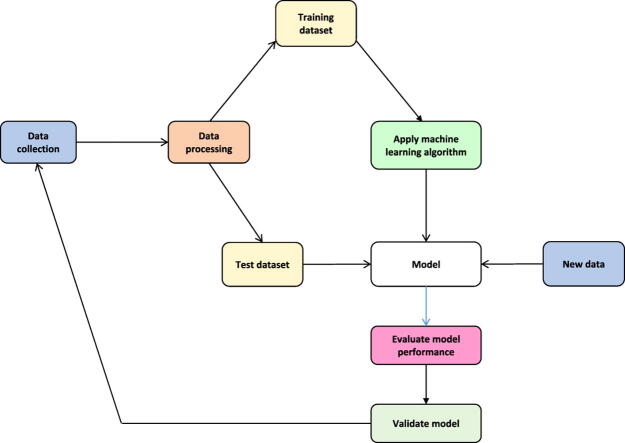

## 1 Introduction

Predictive analytics is applied across many industries, typically for insurance underwriting, credit risk scoring and fraud detection [1], [2], [3]. Both statistical methods and machine learning algorithms are used to create predictive models [4]. In heart failure, machine learning algorithms create risk scores estimating the likelihood of a heart failure diagnosis and the probability of outcomes such as all-cause mortality, cardiac death and hospitalization [5], [6], [7], [8], [9], [10], [11], [12], [13].

Clinicians treating heart failure patients may underestimate or overestimate the risk of complications and may battle with dose titration, failing to reach target dosages when prescribing oral medication such as beta-blockers [14], [15]. Despite these challenges, risk calculators are still not widely used to guide the management of heart failure patients. Most clinicians find risk calculation time consuming and are not convinced of the value of the information derived from predictive models [15], [16]. Moreover, the lack of integration of risk scores predicting heart failure outcomes into management guidelines may diminish clinicians’ confidence when using risk calculators. Also, clinicians may question the integrity of unsupervised machine learning and deep learning methods since algorithms single-handedly select features (*predictors*) without human input.

Machine learning and its subtype, deep learning, have shown an impressive performance in medical image analysis and interpretation [17]. Convolutional neural networks (CNN) were trained to classify chest radiographs as pulmonary tuberculosis (TB) or normal using chest radiographs from 685 patients. The ensemble of CNN’s performed well with an area under the receiver operating characteristic curve (AUC) of 0.99 [17]. These impressive results have resulted in the commercialization of chest x-ray interpretation software [18]. The availability of such software can play a critical role in remote areas with limited or no access to radiologists, as CNN can potentially identify subtle manifestations of TB on chest radiographs, leading to prompt initiation therapy, curbing further transmission of TB. Amid these capabilities, the uptake of machine learning techniques in the healthcare sector remains limited. This systematic review aims to identify models predicting mortality and hospitalization in heart failure patients and discuss factors that restrict the widespread clinical use of risk scores created with machine learning algorithms.

## 2 Methods

### 2.1 Search strategy for identification of relevant studies

A systematic literature search was conducted in accordance with the Preferred Reporting Items for Systematic Reviews and Meta-Analyses (PRISMA) guidelines. Literature searches were conducted in MEDLINE, Google Scholar, Springer Link, Scopus, and Web of Science. The search string contained the following terminology: (Mortality OR Death OR Readmission OR Hospitalization) AND (Machine Learning OR Deep Learning) AND (Heart Failure OR Heart Failure, Diastolic OR Heart Failure, Systolic).

### 2.2 Review methods and selection criteria

Studies reported in languages other than English were not included. A single reviewer screened titles, abstracts and full-text articles and made decisions regarding potential eligibility. Studies were eligible if they reported models predicting all-cause or cardiac mortality or all-cause or heart failure-related hospitalization in heart failure patients. Models included in the study were created using machine learning algorithms and/or deep learning. We did not include studies using solely logistic regression for a classification task. Logistic regression analysis is a machine learning algorithm borrowed from traditional statistics. When logistic regression is used as a machine learning algorithm, the algorithm is initially trained to identify clinical data patterns using a dataset with labelled classes, a process known as supervised learning. After that, the logistic regression algorithm attempts to classify new data into two or more categories based on “posteriori knowledge.”

### 2.3 Data extraction

The following items were extracted: study region, data collection period, sample size, age, gender, cause of heart failure (ischaemic vs non-ischaemic), predictor variables, handling of missing data, internal and external validation, all-cause mortality and cardiovascular death rate, all-cause hospitalization rate and performance metrics (sensitivity, accuracy, AUC or c-statistics and F-score). Summary statistics were generated with STATA MP version 13.0 (StataCorp, Texas).

## 3 Results

### 3.1 The review process

**Fig. 1:**
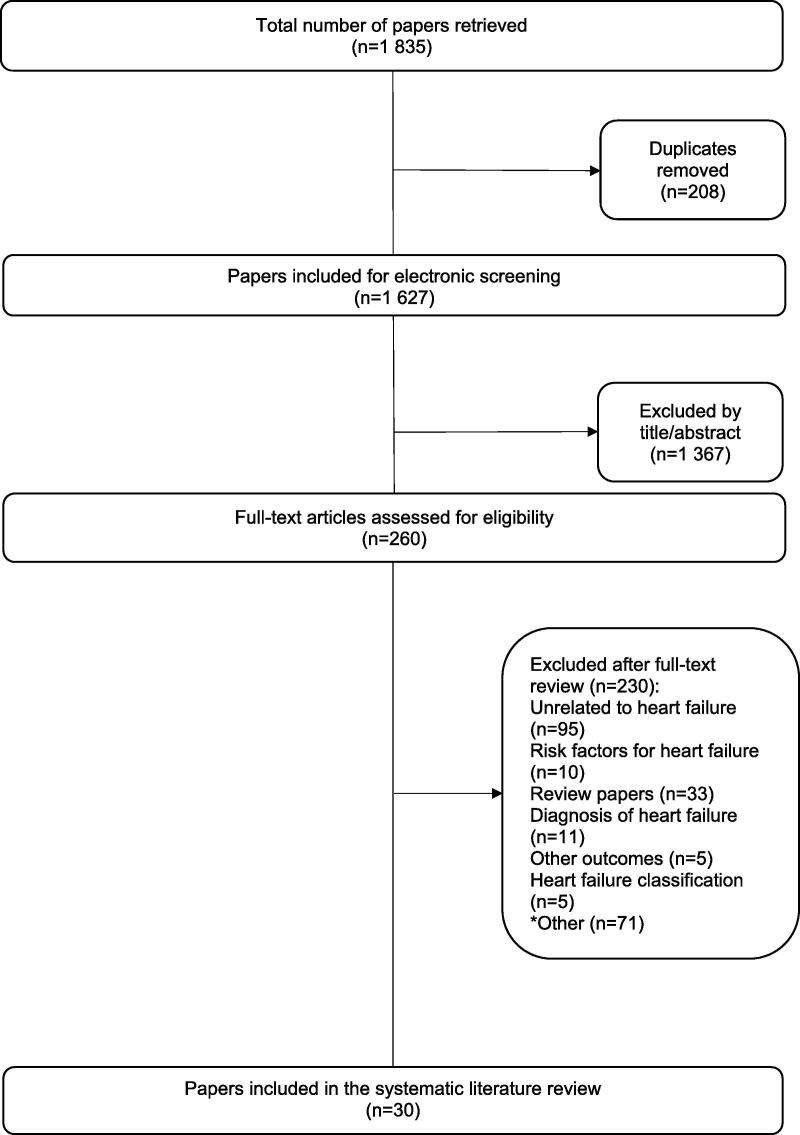
Flow chart of the systematic literature search.

The initial search yielded 1 835 research papers. After screening titles and abstracts, 1 367 did not meet the inclusion criteria. Excluded papers were predominantly theoretical reviews and conference papers in the field of computer science. Two hundred and sixty full-text articles were assessed for eligibility. A further 230 studies were excluded, leaving thirty papers legible for analysis (Fig. 1). Reasons for excluding 230 studies are provided as **supplementary data**.

### 3.2 Characteristics of the included studies

**Fig. 2:**
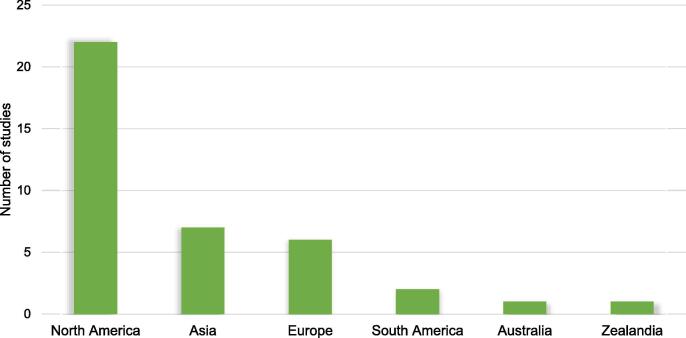
Study population region.

The source of data in the majority of the studies were electronic health records (EHR) (*n* = 16), followed by claims data (*n* = 5), trial data (*n = 3),* registry *(n = 3)* and data obtained from research cohorts (*n = 3)*. Data was collected from hospitalized patients in twelve studies. The sample size in the predictive models ranged between 71 and 716 790, with the smallest sample size used to predict survival in patients with advanced heart failure managed with second-generation ventricular assist devices [19]. Within the 30 studies, twelve studies created models predicting mortality. Another 13 studies predicted hospitalization, and five studies predicted both mortality and hospitalization. The data used to create predictive models was collected between 1993 and 2017 (Table 1**).** Of the 30 included studies, 22 included data originating from North America, seven from Asia and six from Europe. There were no studies conducted in Africa or Middle-East (Fig. 2).

**Table 1:** Characteristics of the included studies.

**Study ID**	**Data collection period**	**No. of patients**	**Setting**	**Data source**	**No. of features**	**Primary outcome assessed**
Adler, E.D (2019) [10]	2006–2017	5 822	Inpatient and outpatient	EHR and Trial	8	All-cause mortality
Ahmad, T (2018) [30]	2000–2012	44 886	Inpatient and outpatient	Registry	8	1-year all-cause mortality
Allam, A (2019) [31]	2013	272 778	Inpatient	Claims dataset	50	30-day all-cause readmission
Angraal, S (2020) [13]	2006–2013	1 767	Inpatient	Trial	26	All-cause mortality and HF hospitalization
Ashfaq, A (2019) [32]	2012–2016	7 655	Inpatient and outpatient	EHR		30-day all-cause readmission
Awan, SE (2019) [33]	2003–2008	10 757	Inpatient and outpatient	EHR	47	30-day HF-related readmission and mortality
Chen, R (2019) [34]	2014–2017	98	Inpatient	Prospective Clinical and MRI	32	Cardiac death, heart transplantation and HF-related hospitalization
Chicco, D (2020) [11]	2015	299	Inpatient	Medical records	13	One year survival
Chirinos, J (2020) [35]	2006–2012	379	Inpatient	Trial	48	Risk of all-cause death or heart failure-related hospital admission
Desai, R.J (2020) [6]	2007–2014	9 502	Inpatient and outpatient	Claims data and EHR	62	All-cause mortality and HF hospitalization, total costs for hospitalization, outpatient visits, and medication
Frizzell, J.D (2017) [36]	2005–2011	56 477	Inpatient	Registry and claims data		All-cause readmission 30-days after discharge
Gleeson, S (2017) [37]	2010–2015	295	Inpatient	Echo database & EHR	291	All-cause mortality and heart failure admissions
Golas, S.B (2018) [12]	2011–2015	11 510	Inpatient and outpatient	EHR	3 512	All-cause 30-day readmission, healthcare utilization cost
Hearn, J (2018) [38]	2001–2017	1 156		EHR and Cardiopulmonary stress test data		All-cause mortality
Hsich, E (2011) [9]	1997–2007	2 231		Cardiopulmonary stress test data	39	All-cause mortality
Jiang, W (2019) [39]	2013–2015	534	Inpatient	EHR	57	30-day readmission
Kourou, K (2016) [19]		71		Pre and post-operative data	48	1-year all-cause mortality
Krumholz, H (2019) [40]	2013–2015	716 790	Inpatient	Claims dataset		All-cause death within 30-days of admission
Kwon, J (2019) [5]	2016–2017	2 165 (training dataset)	Inpatient	Registry		12 and 36-month in-hospital mortality
Liu, W (2020) [41]		303 233 (heart failure)	Inpatient	Readmission database		Admission 3H myocardial infarction, congestive heart failure and pneumonia 30-day readmission
Lorenzoni, G (2019) [7]	2011–2015	380	Inpatient	Research data		Hospitalization among patients with heart failure
Maharaj, S.M (2018) [42]	2015	1 778	Inpatient	EHR	56	30-day readmission
McKinley, D (2019) [20]	2012–2015	132	Inpatient	EHR	29	All-cause readmission within 30-days
Miao, F (2017) [43]	2001–2007	8 059		Public database	32	1-year in-hospital mortality
Nakajima, K (2020) [24]	2005–2016	526		Multicentre database	13	2-year life-threatening arrhythmic events and heart failure death
Shameer, K (2016) [44]		1 068	Inpatient	EHR	4 205	30-day readmission
Shams, I (2015) [45]	2011–2012	1 674	Inpatient	EHR		30-day readmission
Stampehl, M (2020) [46]	2010–2014	206 644	Inpatient	EHR		30-day and one-year post-discharge all-cause mortality
Taslimitehrani, V (2016) [47]	1993–2013	5 044	Inpatient	EHR	43	1,2 and 5-year survival after HF diagnosis
Turgeman, L (2016) [27]	2006–2014	4 840	Inpatient	EHR		Readmission

CVD = cardiovascular disease; EHR = electronic health record; HF = heart failure; MRI = magnetic resonance imaging.

### 3.3 Clinical characteristics of patients with heart failure

The majority of studies reported the patients’ age (93%) and gender (87%). The median age was 72.1 (61.1–76.85) years. Between 14.0 and 83.9% of the extracted studies' participants had ischaemic heart disease (Table 2). In total, 30% of studies mentioned Black patients. Between 0.95% and 100% of the individuals were Black, with one study enrolling only African American males with heart failure [20].

**Table 2:** Characteristics of heart failure patients included in the 30 models predicting mortality and hospitalization.

**First Author (year)**	**Study Region**	**No. of patients**	**% Black**	**Age**	**% male**	**% Hypertension**	**% IHD**
Adler, E.D (2019) [10]	USA and Europe	5 822		60.3			
Ahmad, T (2018) [30]	Europe	44 886		73.2	63		
Allam, A (2019)[31]	USA and Europe	272 778		73 ± 14	51		
Angraal, S (2020)[13]	USA, Canada, Brazil, Argentina, Russia, Georgia	1 767		72 (64–79)	50		
Ashfaq, A (2019) [32]	Europe	7 655		78.8	57		
Awan, SE (2019) [33]	Australia	10 757		82 ± 7.6	49	67	55
Chen, R (2019) [34]	China	98		47 ± 14	79	23	
Chicco, D (2020) [34]	Pakistan	299		40–95*	65		
Chirinos, J (2020) [35]	USA, Canada, Russia	379	7.4	70 (62–77)	53.5	94.5	30.6
Desai, R.J (2020) [6]	USA	9 502	5.1	78 ± 8	45	87.1	22
Frizzell, J.D (2017) [36]	USA	56 477	10	80 (74–86)	45.5	75.7	58
Gleeson, S (2017) [37]	New Zealand	295		62	74	43	
Golas, S.B (2018) [12]	USA	11 510	7.9	75.7 (64–85)	52.8		
Hearn, J (2018) [38]	Canada	1 156		54	74.6		
Hsich, E (2011) [9]	USA	2 231		54 ± 11	73		41
Jiang, W (2019) [39]	USA	534	28	74.8	46		
Kourou, K (2016) [19]	Belgium	71		48.07 ± 14.82	80.3		
Krumholz, H (2019) [40]	USA	716 790	11.3	81.1 ± 8.4	45.6		
Kwon, J (2019) [5]	Asia	2 165		69.8	59.7		
Liu, W (2019) [41]	USA	303 233		72.5	50.9		
Lorenzoni, G (2019) [7]	Italy	380		78 (72–83)	42.9		18.9
Maharaj, S.M (2018) [42]	USA	1 778	0.95	72.3 ± 12.1	97.6		14
McKinley, D (2019) [20]	USA	132	100	59.25	100	91	
Miao, F (2017) [43]	USA	8 059		73.7	54	25	23.2
Nakajima, K (2020) [24]	Japan	526		66 ± 14	72	53	37
Shameer, K (2016) [44]	USA	1 068					
Shams, I (2015) [45]	USA	1 674	70.4	69.9	96		
Stampehl, M (2020) [46]	USA	206 644	12.6	80.5 ± 11.2	38.3	96.5	0.4
Taslimitehrani, V (2016) [47]	USA	5 044		78 ± 10	52	81	70.2
Turgeman, L (2016) [27]	USA	4 840		69.3 ± 11.02		96.5	84.9

Age showed as mean ± standard deviation, median (25th-75th percentile interquartile range) or minimum and maximum value.* IHD: ischaemic heart disease; USA: United States of America.

### 3.4 Machine learning algorithms

**Fig. 3:**
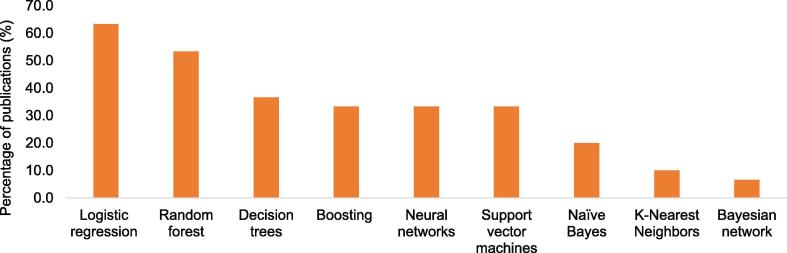
Number of studies using machine learning algorithms.

Only eight (27%) studies used a single algorithm to build a predictive model. Nineteen studies (63%) used logistic regression, 53% random forests, and 36% of studies used decision trees to create predictive models. The rest of the algorithms are depicted in Fig. 3.

### 3.5 Predictors

Twelve (36.4%) studies did not report on the number of predictors or features used. The number of predictors in the identified studies were between 8 and 4 205. Some authors only mentioned the number of predictors and did not list them. Age, gender, diastolic blood pressure, left ventricular ejection fraction (LVEF), estimated glomerular filtration rate, haemoglobin, serum sodium, and blood urea nitrogen were some of the predictors of mortality identified in the extracted studies [10], [11], [13]. Predictors of hospitalization included ischaemic cardiomyopathy, age, LVEF, hypotension, haemoglobin, creatinine, and potassium serum levels [7].

### 3.6 Model development, internal and external validation

When creating a predictive model using machine learning, data is generally partitioned into three or four datasets. In the studies extracted, between 60 and 80% of the data was used for training models, while the rest was used for testing and/or internally validating the models. Although the data on model validation was scanty, external validation was explicitly mentioned in two studies. None of the models were externally validated using data originating from Africa or the Middle-East.

### 3.7 Model performance and evaluation metrics

Parameters used to evaluate model performance were the confusion matrix, reporting sensitivity, specificity, positive and negative predictive value, accuracy, and precision. Most studies also reported the f-score, AUC, concordance statistic (C-statistic), and recall. The minimum and maximum AUC for models predicting mortality were 0.477 and 0.917, and models predicting hospitalization had an AUC between 0.469 and 0.836 (Table 3).

**Table 3:** Performance metrics of algorithms predicting mortality and hospitalization in heart failure.

**Author**	**Algorithms**	**Sensitivity**	**Accuracy**	**AUC (mortality)**	**AUC (Hospitalization)**	**F-score**
Adler, E.D (2019) [10]	Boosted decision trees			0.88 (0.85–0.90)		
Ahmad, T (2018) [30]	Random forest			0.83		
Allam, A (2019) [31]	Recurrent neural network				0.64 (0.640–0.645)	
	Logistic regression l_2_-norm regularization (LASSO)				0.643 (0.640–0.646)	
Angraal, S (2020) [13]	Logistic regression			0.66 (0.62–0.69)	0.73 (0.66–0.80)	
	Logistic regression with LASSO regularization			0.65 (0.61–0.70)	0.73 (0.67–0.79)	
	Gradient descent boosting			0.68 (0.66–0.71)	0.73 (0.69–0.77)	
	Support vector machines (linear kernel)			0.66 (0.60–0.72)	0.72 (0.63–0.81)	
	Random forest			0.72 (0.69–0.75)	0.76 (0.71–0.81)	
Ashfaq, A (2019) [32]	Long Short-Term Memory (LSTM) neural network				0.77	0.51
Awan, SE (2019) [33]	Multi-layer perceptron (MLP)	48.4		0.62		
Chen, R (2019) [34]	Naïve Bayes		0.827	0.855 0.887 0.890 0.877 0.852 0.847 0.705 0.797		
	Naïve Bayes + IG		0.857			
	Random forest		0.817			
	Random forest + IG		0.827			
	Decision trees (bagged)		0.827			
	Decision trees (bagged) + IG		0.816			
	Decision trees (boosted)		0.735			
	Decision trees (boosted) + IG		0.806			
Chicco, D (2020) [11]	Random forest		0.740	0.800		0.547
	Decision tree		0.737	0.681		0.554
	Gradient boosting		0.738	0.754		0.527
	Linear regression		0.730	0.643		0.475
	One rule		0.729	0.637		0.465
	Artificial neural network		0.680	0.559		0.483
	Naïve Bayes		0.696	0.589		0.364
	SVM (radial)		0.690	0.749		0.182
	SVM (linear)		0.684	0.754		0.115
	K-nearest neighbors		0.624	0.493		0.148
Chirinos, J (2020) [35]	Tree-based pipeline optimizer				0.717 (0.643–0.791)	
Desai, R.J (2020) [6]	Logistic regression (traditional)			0.749 (0.729–0.768)	0.738 (0.711–0.766)	
	LASSO			0.750 (0.731–0.769)	0.764 (0.738–0.789)	
	CART			0.700 (0.680–0.721)	0.738 (0.710–0.765)	
	Random forest			0.757 (0.739–0.776)	0.764 (0.738–0.790)	
	GBM			0.767 (0.749–0.786)	0.778 (0.753–0.802)	
Frizzell, J.D (2017) [36]	Random forest				0.607	
	GBM				0.614	
	TAN				0.618	
	LASSO				0.618	
	Logistic regression				0.624	
Gleeson, S (2017) [37]	Decision trees			0.7505		
Golas, S.B (2018) [12]	Logistic regression		0.626		0.664	0.435
	Gradient boosting		0.612		0.650	0.425
	Maxout networks		0.645		0.695	0.454
	Deep unified networks		0.646		0.705	0.464
Hearn, J (2018) [38]	Staged LASSO			0.827 (0.785–0.867)		
	Staged neural network			0.835 (0.795–0.880)		
	LASSO (breath-by-breath)			0.816 (0.767–0.866)		
	Neural network (breath-by-breath)			0.842 (0.794–0.882)		
Hsich, E (2011) [9]	Random survival forest			0.705		
	Cox proportional hazard			0.698		
Jiang, W (2019) [39]	Logistic and beta regression (ML)				0.73	
Kourou, K (2016) [19]	Naïve Bayes	85		0.86		
	Bayesian network	85.9		0.596		
	Adaptive boosting	78		0.74		
	Support vector machines	90		0.74		
	Neural networks	87		0.845		
	Random forest	75		0.65		
Krumholz, H (2019) [40]	Logistic regression (ML)			0.776		
Kwon, J (2019) [5]	Deep learning			0.813 (0.810–0.816)		
	Random forest			0.696 (0.692–0.700)		
	Logistic regression			0.699 (0.695–0.702)		
	Support vector machine			0.636 (0.632–0.640)		
	Bayesian network			0.725 (0.721–0.728)		
Liu, W (2019) [41]	Logistic regression				0.580 (0.578–0.583)	
	Gradient boosting				0.602 (0.599–0.605)	
	Artificial neural networks				0.604 (0.602–0.606)	
Lorenzoni, G (2019) [7]	GLMN	77.8	0.812		0.86	
	Logistic regression	54.7	0.589		0.646	
	CART	44.3	0.635		0.586	
	Random forest	54.9	0.726		0.691	
	Adaptive Boosting	57.3	0.671		0.644	
	Logitboost	66.7	0.625		0.654	
	Support vector machines	57.3	0.699		0.695	
	Artificial neural networks	61.6	0.682		0.677	
Maharaj, S.M (2018) [42]	Boosted tree				0.719	
	Spike and slab regression				0.621	
McKinley, D (2019) [20]	K-nearest neighbor		0.773		0.768	
	K-nearest neighbor (randomized)		0.477		0.469	
	Support vector machines		0.545		0.496	
	Random forest		0.682		0.616	
	Gradient boosting machine		0.614		0.589	
	LASSO		0.614		0.576	
Miao, F (2017) [43]	Random survival forest			0.804		
	Random survival forest (improved)			0.821		
Nakajima, K (2020) [24]	Logistic regression			0.898		
	Random forest			0.917		
	GBT			0.907		
	Support vector machine			0.910		
	Naïve Bayes			0.875		
	k-nearest neighbors			0.854		
Shameer, K (2016) [44]	Naïve Bayes		0.832		0.78	
Shams, I (2015) [45]	Phase type Random forest	91.95			0.836	0.892
	Random forest	88.43			0.802	0.865
	Support vector machine	86.16			0.775	0.857
	Logistic regression	83.40			0.721	0.833
	Artificial neural network	82.39			0.704	0.823
Stampehl, M (2020) [46]	CART					
	Logistic regression					
	Logistic regression (stepwise)			0.74		
Taslimitehrani, V (2016) [47]	CPXR(Log)		0.914			
	Support vector machine		0.75			
	Logistic regression		0.89			
Turgeman, L (2016) [27]	Naïve Bayes	48.9			0.676	
	Logistic regression	28.1			0.699	
	Neural network	8.9			0.639	
	Support vector machine	23.0			0.643	
	C5 (ensemble model)	43.5			0.693	
	CART (boosted)	22.6			0.556	
	CART (bagged)	9.0			0.579	
	CHAID Decision trees (boosted)	30.3			0.691	
	CHAID Decision trees (bagged)	10.5			0.707	
	Quest decision tree (boosted)	20.3			0.487	
	Quest decision tree (bagged)	7.2			0.579	
	Naïve network + Logistic regression	38.2			0.653	
	Naïve network + Neural network	26.3			0.635	
	Naïve network + SVM	35.8			0.649	
	Logistic regression + Neural network	16.8			0.59	
	Logistic regression + SVM	26.2			0.607	
	Neural network + SVM	16.5			0.577	

AUC: area under the receiver operating characteristic curve; CART: classification and regression tree; CPXR: contrast pattern aided logistic regression; GBM: gradient-boosted model; HR: hazard ratio; IG: information gain; LASSO: least absolute shrinkage and selection operator; ML: machine learning; SVM: support vector machine; TAN: tree augmented Bayesian network. The AUC is displayed under both the mortality and hospitalization column if the authors did not specify the outcome predicted.

## 4 Discussion

This systematic review highlights several factors that restrict the use of risk scores created with machine learning algorithms in the clinical setting. The existence of clinical information with prognostic significance such as the New York Heart Association functional class in the free-text format in EHR systems may result in models with low predictive abilities if such critical data is omitted when building predictive models. Fortunately, newer emerging techniques such as bidirectional long short-term memory with a conditional random fields layer have been introduced to remedy the problem of free-text in EHR [21], [22].

Risk scores derived from heart failure patients residing in North America or Europe may not be suitable for application in low and middle-income countries (LMIC). In high income countries (HIC), the predominant cause of heart failure is ischaemic heart disease (IHD), whereas, in sub-Saharan Africa, hypertension is still the leading cause of heart failure [23]. Also, healthcare services' availability and efficiency differ significantly between countries, suggesting that algorithms trained using data from HIC should be retrained using local data before adopting risk calculators.

Despite the endemicity of heart failure in LMIC, risk scores derived from patients residing in LMIC are scanty or non-existent. The lack of EHR systems, registries, and pooled data from multicentre studies is responsible for the absence of risk scores derived from patients in LMIC. If digital structured health data were available in LMIC, models predicting outcomes could be created instead of extrapolating from studies conducted in HIC. The absence of structured health data in LMIC resulted in the underrepresentation of this population in the training and test datasets included in this systematic review.

The AUC was one of the most commonly reported performance metric in the extracted studies. The highest AUC for models predicting mortality was 0.92, achieved by the random forest algorithm in a study by Nakajima et al., where both clinical and physiological imaging data were used to train algorithms [24]. A model with an AUC equal to or below 0.50 is unable to discriminate between classes. One might as well toss a coin when making predictions. Some of the reasons for the modest performance metrics demonstrated by machine learning algorithms include a training dataset with excessive missing data or few predictors, absence of ongoing partnership between clinicians and data scientists and class imbalance. In most instances, when handling healthcare data, the negative class tends to outnumber positive classes. The learning environment is rendered unfavourable since there are fewer positive observations or patterns for an algorithm to learn from. For example, when predicting mortality, the class with patients that demised is frequently smaller than the class with alive patients.

Models with perfect precision and recall have an F-measure, also known as the F-Score or F1 Score, equal to one [25]. Sensitivity, also known as recall, measures a proportion of positive classes accurately classified as positive [26]. Machine learning algorithms in the extracted studies had a sensitivity rate between 7.2 and 91.9%. The low sensitivity, reported by Turgeman and May, improved to 43.5% when they used an ensemble method to combine multiple predictive models to produce a single model [27].

Although the random forest algorithm appeared to have the highest predictive abilities in most studies, one cannot conclude that it should be the algorithm of choice whenever one attempts to create a predictive model. The random forest algorithm's main advantage is that it is an ensemble-based classifier that takes random samples of data, exposing them to multiple decision tree algorithms. Decision trees are intuitive and interpretable and can immediately suggest why a patient is stratified into a high-risk category, hence guiding subsequent risk reduction interventions. The interpretability of decision trees is a significant advantage in contrast to deep learning methodologies such as artificial neural networks with a “black box” nature. Once random samples of data have been exposed to multiple decision tree algorithms, the decision trees' ensemble identifies the class with the highest number of votes when making predictions. Random forests also perform well in large datasets with missing data, a common finding when handling healthcare data, and can rank features (*predictors*) in the order of importance, based on predictive powers [28].

Predictors of mortality identified by machine learning algorithms in the extracted studies were explainable and included features such as the LVEF, hypotension, age and blood urea nitrogen levels. Whether these predictors should be considered significant risk factors for all heart failure, irrespective of genetic makeup, is debatable. The youngest patient in the studies reviewed was 40 years old, but most of the patients included in the predictive models were significantly older, with a median age of 72 years. Risk scores derived from older patients may reduce the applicability of the existing risk calculators in the sub-Saharan African (SSA) context, considering that patients with heart failure in SSA are generally a decade younger [29].

Geographically unique heart failure aetiologies and diverse clinical presentations call for predictive models that incorporate genomic, clinical and imaging data. We recommend that clinicians treating heart failure patients focus on establishing structured EHR systems and comparing outcomes such as mortality and hospitalization in patients managed with and without risk scores. Clinicians without access to EHR systems should carefully study the cohort used to create risk scores before implementing risk scores in their clinical practice.

## 5 Limitations

This systematic literature review has several limitations. The systematic literature search was conducted by a single reviewer, predisposing the review to selection bias. We only included original research studies published after 2009. The rationale for including studies published in the past 11 years was to avoid including studies where rule-based expert systems were used instead of newer machine learning techniques. Although the data used to create predictive models was grossly heterogeneous, a meta-analytic component as part of the review would have provided a broader perspective on machine learning algorithms' performance metrics when predicting heart failure patient outcomes.

## 6 Conclusion

The variation in the aetiologies of heart failure, limited access to structured health data, distrust in machine learning techniques among clinicians and the modest accuracy of predictive models are some of the factors precluding the widespread use of machine learning derived risk calculators.

## 7 Grant support

The study did not receive financial support. The primary author Dr Dineo Mpanya is a full-time PhD Clinical Research fellow in the Division of Cardiology, Department of Internal Medicine at the University of the Witwatersrand. Her PhD is funded by the Professor Bongani Mayosi Netcare Clinical Scholarship, the Discovery Academic Fellowship *(Grant No. 039023)*, the Carnegie Corporation of New York (*Grant No. b8749*) and the South African Heart Association.

## Declaration of Competing Interest

All authors take responsibility for all aspects of the reliability and freedom from bias of the data presented and their discussed interpretation.
