# Supplementary material for: Predicting mortality and hospitalization in heart failure using machine learning: A systematic literature review
Source: Int J Cardiol Heart Vasc. 2021 Apr 12;34:100773. doi: 10.1016/j.ijcha.2021.100773 (PMC8065274; doi:10.1016/j.ijcha.2021.100773)
Supplement: Supplementary Data 1 [file mmc1.docx]

Excluded studies

Original research studies unrelated to heart failure (n=95), studies designed for the identification of risk factors for heart failure (n=10), prediction of outcomes other than mortality or rehospitalisation (n=5), narrative review paper related to heart failure (n=10), review paper unrelated to heart failure (n=23), diagnosis of heart failure using an electrocardiogram (n=2), evaluation of response of cardiac resynchronisation therapy (n=1), heart failure validation study (n=1), evaluation of predictors (n=1), limited or no clinical data related to heart failure patients (n=8), abstract/poster only (n=14), study protocol (n=1), heart failure classification (n=5), cardiovascular disease classification (n=3), rehospitalisation of all conditions with no isolated data on heart failure patients (n=12), detection of heart failure (n=9), predicting heart failure (n=6), home monitoring of heart failure (n=1), distinguishing heart failure patients from normal subjects (n=3), tele monitoring in heart failure (n=2), regression analysis with traditional statistical methods only (n=12), models created with Cox proportional hazard for survival analysis in heart failure (n=4), Bowtie model for palliative care (n=1), study using mathematical formula not classified as a machine learning algorithm (n=1).
